# Circulating Biomarkers as a Window into the Relationship between Sarcopenia and Diabetes: Implications for Effective Management

**DOI:** 10.2174/0115733998349193250207045239

**Published:** 2025-02-13

**Authors:** Ali Tajik, Vahid Mahdavizadeh, Farzin Hadizadeh, Neda Shakour

**Affiliations:** 1 Faculty of Medicine, Mashhad University of Medical Sciences, Mashhad, Iran;; 2 Clinical Research Development Unit, Faculty of Medicine, Ghaem Hospital, Mashhad University of Medical Sciences, Mashhad, Iran;; 3 Biotechnology Research Center, Pharmaceutical Technology Institute, Mashhad University of Medical Sciences, Mashhad, Iran;; 4 Department of Medicinal Chemistry, School of Pharmacy, Mashhad University of Medical Sciences, Mashhad, Iran

**Keywords:** Aging, sarcopenia, glucose uptake, diabetes, circulating biomarkers, HbA1c, insulin

## Abstract

**Introduction/Aims:**

This study aims to investigate the relationship between sarcopenia and circulating biomarkers in diabetes, with a focus on early detection and effective management strategies.

**Methods:**

A literature review was conducted using the ScienceDirect, Scopus, PubMed, and Web of Science databases up to December 2024. Key search terms included “diabetes,” “sarcopenia,” “HbA1c”, “glucose,” “insulin,” and specific biomarkers such as inflammatory markers, adipokines, and myokines.

**Results and Discussion:**

Aging is associated with a decline in organ and bodily system functionality, with sarcopenia being particularly prominent due to its progressive loss of muscle mass and function. This condition increases health risks and mortality in the elderly. Muscles, as the primary consumers of glucose, play a crucial role in glucose uptake; reduced mass can exacerbate insulin resistance. Sarcopenia and diabetes share common pathophysiological mechanisms, including insulin resistance, inflammation, and vascular complications. Circulating biomarkers, crucial for diabetes management, may offer insights into the early stages of sarcopenia.

**Conclusion:**

The complex relationship between sarcopenia and diabetes, influenced by shared pathophysiological pathways, presents challenges in geriatric healthcare. Circulating biomarkers hold promise for early detection and monitoring of sarcopenia, potentially enhancing patient outcomes and quality of life. Further research is necessary to validate these connections and develop targeted treatments for individuals affected by these conditions.

## 1 INTRODUCTION

Sarcopenia is a condition commonly seen in older individuals, characterized by a gradual reduction in both muscle mass and function. This condition has been linked to various negative health outcomes, including fractures, a decline in functional abilities, and increased mortality rates [1]. The prevalence of sarcopenia ranges from 11% to 50% in individuals aged 80 and above, with rates reported to be as high as 29% in community healthcare settings for older adults [2]. Sarcopenia can also occur in mid-life [1] and is prevalent among specific populations, such as individuals with cancer [3], kidney dysfunction [4], liver disease [5], and metabolic disorders like diabetes mellitus (DM) [6].

Notably, skeletal muscle constitutes approximately 50% of the body weight, and during periods of rest, roughly 15% of the circulating blood volume is directed to the skeletal muscle [7]. In the context of diabetes, skeletal muscle plays a pivotal role in glucose metabolism, with insulin facilitating the uptake of over 80% of glucose in peripheral tissues [8-11], primarily by skeletal muscles. Individuals with long-standing T2DM are prone to losing muscle mass, particularly in the lower extremities, and experiencing a decline in strength. This could be due to a decrease in predominantly oxidative fiber types within the muscles [12]. This decrease in skeletal muscle mass due to sarcopenia leads to reduced glucose uptake by insulin, contributing to insulin resistance (IR) [13]. Consequently, individuals with type 2 diabetes mellitus (T2DM) experience impaired glucose uptake within skeletal muscles [14, 15]. Recent research highlights the beneficial effects of moderate exercise in preventing and managing type 2 diabetes by enhancing energy expenditure, reducing obesity, and promoting glucose metabolism [16-23].

Recent studies proposed various mechanisms underlying the development of sarcopenia in diabetic patients. Fluctuations in glucose levels can impair endothelial cell function and perpetuate chronic inflammation through oxidative stress pathways, ultimately hindering muscle growth and accelerating protein degradation [24, 25]. Interestingly, poorly controlled glucose fluctuations may induce oxidative stress more prominently than sustained hyperglycemia alone [26]. Furthermore, these fluctuations can activate AGEs, contributing to the cross-linking and degradation of muscle proteins, thereby increasing muscle stiffness and susceptibility to injury [27]. AGEs have the potential to augment oxidative stress, and inflammatory cytokines, and facilitate the formation of cross-links and disruptions of muscular proteins among the elderly population [27, 28].

The relationship between sarcopenia and cardiovascular diseases (CVDs) is increasingly recognized as a critical area of study, especially in the context of diabetes. A reduction in muscle mass may result in CVDs *via* shared pathogenetic pathways, including hormonal alterations, malnutrition, and physical inactivity, which interact with one another [29]. Meanwhile, conditions such as CVDs, particularly heart failure (HF), can expedite muscle degradation *via* systemic inflammation and diminished blood and oxygen supply to muscles, establishing a bidirectional relationship between sarcopenia and CVDs [30]. This highlights the crucial role of muscle health in both metabolic and cardiovascular diseases, with significant implications for patient outcomes.

The correlation between T2DM and sarcopenia involves a reciprocal relationship, where the presence of one condition increases the likelihood of the other emerging. Several key factors, including insulin resistance, inflammation, accumulation of AGEs, heightened oxidative stress, and vascular complications, influence various aspects of muscle health. Conversely, compromised muscle health can also contribute to the onset and progression of T2DM [31]. Circulating biomarkers play a crucial role in identifying and monitoring diabetes, offering cost-effective and efficient management strategies for patients [32, 33]. Recent research has been directed toward investigating the association between biomarkers and the manifestation of muscle weakness in individuals with diabetes. These biomarkers encompass a diverse range of molecules, such as inflammatory cytokines, oxidative stress markers, adipokines, and myokines, reflecting the intricate interplay of biological processes involved [34-36]. Understanding the relationship between these biomarkers and muscle health in the context of diabetes is essential for elucidating underlying mechanisms and developing targeted interventions. This study endeavors to investigate the association between sarcopenia and circulating biomarkers in diabetes, with a primary objective of enhancing overall quality of life and, developing novel strategies for preserving muscle health and addressing metabolic imbalances in affected individuals.

## 2 MATERIALS AND METHODS

This study involved searching ScienceDirect, Scopus, PubMed, and Web of Science for English language articles published up to April 2024. The key terms included “diabetes” and “sarcopenia,” or “muscle wasting,” and “Hemoglobin A1c”, or “HbA1c” or “glycosylated hemoglobin” and “glucose,” and “insulin,” and “C peptide,” or “adiponectin,” or “leptin,” and “cholesterol,” and “triglyceride,” and “inflammatory markers,” and “tumor necrosis factor-α,” or “TNF-α,” and “interleukin-6”, or “IL-6”, and “C-reactive protein,” or “CRP,” and “ketone,” and “blood urea nitrogen,” or “BUN,” and “creatinine,” and “urinary L-FABP,” and “cystatin C,” and “gamma-glutamyl transpeptidase” or “GGT,” and “resistin,” and “glucagon like peptide-1”, or “GLP-1”.

## 3 AN OVERVIEW OF DIABETES-RELATED CIRCULATING BIOMARKERS ON SARCOPENIA PATHOGENESIS

### 3.1 Glucose

Normally, plasma glucose concentrations are typically regulated within a relatively narrow range throughout the day despite significant fluctuations in the delivery and removal of glucose from the circulation [37]. Maintaining blood glucose levels within a tight range is a critical physiological function that involves multiple metabolic pathways and various cell types, such as pancreatic cells, hepatocytes, and muscle cells [38]. Under hormonal regulation, hepatocytes adjust glucose storage or production in response to feeding or fasting conditions. Accurate measurement of blood glucose levels is essential in the clinical diagnosis and monitoring of DM, as well as in screening and epidemiological studies of glucose tolerance. Reproducible methods are crucial in practical clinical settings to ensure the accuracy of monitoring patients' circulating glucose levels [39]. Several methods, such as fasting plasma glucose (FPG), fasting plasma insulin (FPI), homeostatic model assessment (HOMA), meal tolerance test (MTT), oral glucose tolerance test (OGTT), intraperitoneal insulin sensitivity test (IPIST), and intraperitoneal glucose tolerance test (IPGTT), are used to measure blood glucose [40].

In the fasting state, glucagon prevents hypoglycemia by stimulating gluconeogenesis and glycogenolysis, leading to hepatic glucose release. Postprandially, insulin prevents hyperglycemia by inhibiting hepatic gluconeogenesis and glycogenolysis while promoting hepatic glycogen synthesis. Elevated blood glucose levels can drive glucose into the polyol pathway, increasing sorbitol production, which is subsequently converted to fructose. Metabolites generated from this pathway diminish cellular antioxidant defense, foster the generation of precursors for AGEs, and trigger activation of the protein kinase C pathway [41]. These processes heighten oxidative stress and inflammation, and disrupt muscular proteins [27, 28].

Overall, glucose fluctuations are associated with various disorders, including diminished sleep quality, increased depression and anxiety, inflammation, neuronal apoptosis, cardiovascular diseases, and reduced muscle mass [42-45].

#### 3.1.1 Correlation Between Sarcopenia and Glucose

Currently, there is limited data on the relationship between blood glucose fluctuation and sarcopenia in patients with T2DM. However, there are studies investigating the association between sarcopenia and blood glucose levels. Ogama *et al.* proposed the hypothesis that irregular glucose profiles would be correlated with changes in muscle and physical dysfunction. To explore this, they conducted a study examining the presence of sarcopenia in diabetes patients with and without cognitive impairment and explored the connection between glucose changes and sarcopenia. Daily glucose levels were monitored through self-monitoring of blood glucose, with a specific focus on the effects of glucose fluctuations, postprandial hyperglycemia, and the frequency of hypoglycemia on sarcopenia. Diabetes patients with cognitive impairment showed a high prevalence of sarcopenia, and glucose fluctuations were found to be independently linked to sarcopenia. The findings suggested that glucose fluctuations were significantly associated with decreased muscle mass, weak grip strength, and slow walking speed [46]. In another study, Lin *et al.* (11) demonstrated that patients with sarcopenia showed greater glucose fluctuations than those without sarcopenia. They conducted a study analyzing daily glucose fluctuations and islet function in 482 patients with T2DM over 36 months. The result showed that patients who developed sarcopenia had poorer glucose regulation and insufficient insulin secretion. In addition to mean glucose level, some studies have shown that fluctuating hyperglycemia has more harmful effects on sarcopenia than chronic hyperglycemia. Ma *et al.* [47] conducted a study to investigate the relationship between blood glucose fluctuation and sarcopenia in elderly patients with T2DM. The data indicated that the glucose fluctuation was more pronounced in the sarcopenia group compared to the non-sarcopenia group. Additionally, the incidence rate of sarcopenia decreased with a higher Time in Range (TIR), which represents the percentage of time that blood glucose levels are within the target range of 3.9 to 10.0 mmol/L in a 24-hour period. Similarly, Shi *et al.* [48] also found that low muscle mass was more common in patients with higher glucose fluctuations.

Furthermore, previous studies have shown that glucose-lowering medications for T2DM, such as TZDs, GLP-1 RAs, and SGLT2i, had the potential to improve sarcopenia by enhancing glucose regulation and reducing vascular complications. However, certain agents like metformin and insulin have been found to increase the risk of sarcopenia by affecting muscle protein balance and worsening insulin resistance [49-52] (Fig. **1**).

### 3.2 Hemoglobin A1c (HbA1c)

Hemoglobin A1c (HbA1c) was first recognized as an anomalous hemoglobin variant in individuals with diabetes by Rahbar *et al.* in the 1960s [53]. Its concentration is widely acknowledged as the most precise reflection of average blood sugar levels over recent weeks to months. Frequent monitoring of HbA1c levels is pivotal for accurately documenting glucose metabolism control in diabetic patients. Additionally, it provides a valuable tool for assessing the correlation between carbohydrate control and the onset of complications [54]. Clinical labs use three primary methods for HbA1c testing: Chromatography-based HPLC assay, Antibody-based immunoassay, and Enzyme-based enzymatic assay [55, 56]. For an HbA1c test to be classified as normal or in the non-diabetic range, the value must be below 5.7%. Anyone with an HbA1c value of 5.7% to 6.4% is considered to be prediabetic, while diabetes can be diagnosed with a HbA1c of 6.5% or higher [55].

The reliability of the HbA1c test can be compromised in certain cases. Individuals with conditions such as sickle cell anemia, thalassemia, anemia, kidney failure, liver disease, or those receiving blood transfusions may experience inaccurate results because of the expended lifespan of red blood cells. Therefore, interpreting HbA1c levels in these individuals requires careful attention. It is essential to verify the results with plasma glucose samples to ensure an accurate diagnosis and effective management of diabetes [57].

Furthermore, a falsely low HbA1c value may arise from various factors, encompassing high altitude, pregnancy, hemorrhage, blood transfusion, erythropoietin administration, iron supplementation [58], hemolytic anemia, chronic kidney failure, liver cirrhosis, alcoholism, sickle cell anemia, and spherocytosis [59]. Additionally, the influence of vitamin C supplementation on HbA1c levels is noteworthy, potentially resulting in either an increase or decrease based on the measurement method employed [60]. Conversely, a falsely high HbA1c result may be attributed to conditions such as iron deficiency state [61], infection-induced anemia, or tumor-induced anemia. Furthermore, hemoglobinopathies like thalassemia and B12 deficiency can contribute to falsely elevated HbA1c levels [62]. Other factors linked to elevated HbA1c levels include hypertriglyceridemia, organ transplantation, and hyperglycation in specific ethnic groups. Moreover, certain medications, including immunosuppressants and protease inhibitors, may also lead to inaccurately high HbA1c readings [57, 63]. Understanding these influences is essential for accurate interpretation of HbA1c levels and effective management of diabetes.

Elevated levels of HbA1c have been linked to various diabetic complications, including microvascular complications such as retinopathy, nephropathy, and neuropathy. Moreover, research suggests that high HbA1c levels are associated with reduced muscle strength and an increased risk of sarcopenia [27].

#### 3.2.1 Correlation Between Sarcopenia and HbA1c

Numerous studies have explored the relationship between glycemic control and HbA1c levels in diabetic patients with sarcopenia. Some research suggested that higher levels of glycated HbA1c are associated with declines in muscle quality, mass, and physical performance [64, 65]. Studies by Kalyani *et al.* have shown that elevated HbA1c is linked to decreased muscle strength and aging [66]. Additionally, Sugimoto *et al.* [67] identified a close association between elevated HbA1c and loss of muscle mass among non-obese patients with T2DM. Their research found a stronger correlation between elevated HbA1c levels and the decline in muscle mass in individuals with T2D and a Body Mass Index (BMI)<25/m^2^ compared to reductions in grip strength or walking speed. Moreover, there is evidence to suggest that inadequate glycemic control in the elderly may exacerbate age-related deterioration in muscle quality, especially in the lower extremities [68]. On the other hand, another study suggests that although patients with sarcopenia may have had diabetes for a longer duration, their HbA1c levels are similar to those seen in individuals without sarcopenia [69]. Mori *et al.* [70] indicated that HbA1c was not linked to low muscle mass, grip strength and knee extension strength in their study. Despite the controversy surrounding the association between serum HbA1c levels and sarcopenia, longitudinal studies have demonstrated that insulin therapy and reducing HbA1c concentrations can effectively slow the progression of sarcopenia in elderly individuals diagnosed with T2D [71, 72]. The serum levels of HbA1c and other diabetes-related circulating biomarkers in sarcopenic patients with type 2 diabetes mellitus are detailed in Table **1** [26, 27, 64, 65, 67, 73-98], while Table **2** provides reference ranges for these biomarkers in healthy populations [99-102].

### 3.3 Insulin

Insulin is a peptide hormone secreted by the beta cells of the pancreatic islets of Langerhans. It helps maintain normal blood glucose levels by aiding in cellular glucose uptake, regulating carbohydrate, lipid, and protein metabolism and promoting cell division and growth through its mitogenic effects [103]. Immune-based assays like enzyme-linked immunosorbent assay (ELISA), chemiluminescence immunoassay (CLIA), radioimmunoassay (RIA), and on-chip immunoassay are effective in detecting insulin, with minimal interference and high efficiency. Chromatographic assays, such as high-performance liquid chromatography coupled with ultraviolet detection (HPLC-UV), micellar electrokinetic capillary chromatography (MECC), and liquid chromatography with tandem mass spectrometry (LC-MS/MS), are highly sensitive and capable of simultaneously detecting insulin and its analogues [104]. Insulin secretion from islet cells into the portal veins follows a distinct pulsatile pattern, with concentration oscillations occurring every 5 to 15 minutes. These oscillations are a result of synchronized secretory bursts from multiple islets. Coordination between islets and the pancreas is essential for generating these common secretory bursts, with the pulsatile mechanism playing a dominant role in insulin release [105]. When stimulated, such as by glucose, insulin secretion is typically biphasic, starting with a rapid, followed by a more sustained release of the hormone [106].

Insulin initiates its activity by binding to a glycoprotein receptor on the cell's surface. This receptor is composed of an α-subunit, which binds the hormone, and a β-subunit, which is an insulin-stimulated, tyrosine-specific protein kinase. Activation of this kinase is believed to generate a signal that ultimately influences insulin's effects on glucose, lipid, and protein metabolism [107]. In all cell types, activated insulin receptor triggers downstream metabolic signaling by initially recruiting phosphotyrosine-binding scaffold proteins, which in then activate downstream effectors [108]. Insulin's effects are divers and widespread, varying based on the physiological function of the tissues and organs involved, as well as their reliance on insulin for metabolic processes. Tissues that rely on insulin for intracellular glucose transport, such as adipose tissue and muscle, are considered insulin-dependent [103]. It is useful to categorize insulin's effects into two main groups: growth-promoting and metabolic [109]. Insulin's metabolic effects are typically observed at low hormone concentrations and manifest quickly after exposure to the hormone. Conversely, the growth-promoting of insulin is usually only seen at high insulin concentrations and may take hours or days to become evident [107].

Physiologically, at the whole-body level, the actions of insulin are influenced by the interplay of other hormones [103]. Insulin, as the dominant hormone driving metabolic processes in the fed state, acts in tandem with growth hormone and IGF-1 (insulin-like growth factor 1). Growth hormone is secreted in response to insulin, among other stimuli, to prevent insulin-induced hypoglycemia [103]. Insulin is an anabolic hormone that increases protein synthesis in the muscle and promotes glucose uptake and glycogen synthesis [49] (Fig. **2**). A subnormal biological response to a specific insulin concentration is known as insulin resistance. The range of clinical manifestations associated with insulin resistance is extensive, encompassing patients with diabetes mellitus who require insulin and continue to have hyperglycemia despite large doses of exogenous insulin. Additionally, there are individuals with severe insulin resistance who can sustain nearly normal glucose levels through significantly heightened production of internal insulin [110]. Loss of muscle mass is closely linked to insulin resistance and metabolic syndrome [13]. Skeletal muscle loss and accumulation of intramuscular fat are associated with a variety of pathologies due to a combination of factors, including oxidative stress, inflammatory cytokines, mitochondrial dysfunction, insulin resistance, and inactivity [111].

The mechanisms of insulin's anabolic effects on skeletal muscle are well recognized, involving pathways such as phosphoinositide3-kinase, phosphoinositol triphosphate, protein kinase B/AKT, TBC1 domain family member 4 (TBC1D4) and TBC1 domain family member 1 (TBC1D1). These pathways, in addition to triggering protein synthesis, also facilitate the movement of glucose transporter type 4 (GLUT4) containing vesicles to the plasma membrane [112]. AKT also activates mTOR, suppressing protein synthesis while simultaneously inhibiting Fork head box O (FOXO) transcription factor activity, thereby reducing the expression of E3 ubiquitin ligases that are responsible for muscle atrophy [113]. In the context of T2DM and associated insulin resistance, these pathways gradually deteriorate, resulting in reduced protein synthesis and increased protein degradation, resulting in muscle mass and strength loss [114]. Additionally, insulin resistance contributes to reduced muscle mass and contractility by increasing levels of inflammatory cytokines and myostatin expression, and inhibiting skeletal muscle glucose uptake and blood flow [115].

#### 3.3.1 Correlation between Sarcopenia and Insulin

The relationship between insulin resistance and sarcopenia has been receiving a lot of attention. Decreased insulin actions caused by insulin resistance and/or depletion seem to play a role in worsening muscle dysfunction and sarcopenia [112]. Insulin resistance is the main mechanism behind sarcopenic obesity and is also linked to various cardiometabolic disorders, such as cardiovascular disease, type 2 diabetes mellitus, and non-alcoholic fatty liver disease [116]. The accumulation of fat within muscle fibers triggers an inflammatory response and oxidative stress, which disrupts mitochondrial activity, reduces the effectiveness of insulin signaling pathways, and ultimately leads to muscle atrophy [117, 118]. The interplay between myokines (proteins released from skeletal muscle cells) and adipokines (hormones mainly produced by healthy fat tissue with insulin-sensitizing effects) creates a negative feedback loop. That worsens sarcopenic obesity and insulin resistance [119].

Koo *et al.* [120] demonstrated a significant relationship between diabetes and sarcopenia, particularly in the elderly cohort rather than middle-aged individuals. Specifically, elderly subjects exhibited heightened insulin resistance, while younger participants displayed diminished insulin secretion capacity. These findings highlight a distinct pattern of type 2 diabetes progression across age groups, emphasizing a stronger correlation between sarcopenia and insulin resistance compared to insulin depletion. Furthermore, a research team [121] investigated the impact of hyperinsulinemia on muscle protein metabolism across different age groups. Their findings revealed a significant reduction in the overall protein anabolic response to insulin with advancing age. This attenuation appears to be mediated by the diminished vasodilatory response of aged muscle to insulin, which may impede the efficient delivery and utilization of nutrients necessary for muscle growth during the aging process. On the contrary, a study [122] revealed a strong correlation between sarcopenia and insulin resistance across different age groups, highlighting the pivotal role of diminished muscle mass as an independent risk factor for metabolic disorders. Kim *et al.* [123] also demonstrated that sarcopenic obesity, assessed through dual-energy X-ray absorptiometry and computed tomography, is significantly and independently associated with insulin resistance in both male and female populations.

Moreover, current clinical evidence suggests that administering insulin could reduce the progression of sarcopenia in patients with T2DM. In a retrospective observational study [94], insulin administration was observed to have a protective effect against the decline of the skeletal muscle index (SMI), specifically in the lower extremities. However, this protective effect was not observed in the upper extremities. In another study, Sugimoto *et al.* [124] demonstrated that in Japanese patients with type 2 diabetes, there was a significant association between correcting poor glycemic control, using insulin, and an increase in skeletal muscle mass or gait speed. This association was independent of potential covariates, such as exercise habits.

### 3.4 C-peptide

C-peptide is not a product of therapeutically administered exogenous insulin but rather serves as a valuable indicator of insulin secretion. It is cleaved from proinsulin, stored in secretory granules, and released into the bloodstream in amounts equimolar to endogenous insulin [125]. Estimation methods include urinary, unstimulated, and stimulated serum sampling [126]. Modern assays like Radioimmunoassay (RIA), ELISA, CLIA, Automated immunoassay analyzer AIA-600II, Time-resolved fluoroimmunoassay, and In-house immunochemiluminometric assay are used to detect levels of c-peptide [127]. Assessing insulin secretion *via* C-peptide measurement is crucial for tailoring treatment strategies for Type 1 and Type 2 diabetes [126]. C-peptide levels are closely associated with the type and duration of diabetes, especially concentrations below 0.2 nmol/l, indicating a diagnosis of type 1 diabetes mellitus (T1DM). Additionally, C-peptide levels may correlate with the development of microvascular and macrovascular complications, as well as the likelihood of requiring insulin therapy in the future. Moreover, they can also serve as predictors of individual responses to therapy [128].

The exact mechanisms of C-peptide's cellular interactions are not fully understood. Using fluorescence correlation spectroscopy, researchers have demonstrated the stereospecific binding of C-peptide to various cell types, including renal tubular cells, endothelial cells, skin fibroblasts, mesangial cells, and neuroblastoma cells [129]. It is important to highlight that C-peptide binding does not exhibit cross-reactivity with insulin, IGF-1, or insulin-like growth factor 2 (IGF-2) [130]. Direct interaction with lipid cell membrane components is unlikely. However, the specific cellular binding structure responsible for C-peptide interactions remains elusive, and a dedicated receptor has yet to be identified [130].

In the musculoskeletal system, C-peptide has a modulatory effect on muscle mass and glucose homeostasis through the pAMPK (Phosphorylated AMP-activated protein kinase) pathway [131]. AMP-activated protein kinase (AMPK) is a significant enzyme that plays a crucial role in regulating skeletal muscle mass and glucose uptake in muscles [132]. It has been shown that the activation of AMPK during exercise not only regulates nutrient supply but also stimulates the formation of mitochondria in human skeletal muscle [133, 134]. Furthermore, AMPK-mediated modulation of FoxOs contributes to activating the autophagy systems in skeletal muscle cells [135]. FOXOs also stimulate muscle-specific E3 ubiquitin ligases such as Atrogin-1 (Fbxo32) and Murf-1 (Trim63), which play a pivotal role in sarcomeric breakdown and the suppression of protein synthesis in skeletal muscle, by attaching poly-ubiquitin chains to lysine residues of proteins [136, 137]. It has been proposed that C-peptide may inhibit the ubiquitin-proteasome system (UPS). Activation of the UPS typically results in protein degradation and reduction in muscle mass [138], a process implicated in preventing muscle wasting or cachexia in Type 1 diabetes (T1DM) [131].

The process of C-peptide signal transduction involves the phosphorylation of extracellular signal-regulated kinase 1/2 (ERK1/2) and simultaneous activation of Akt (also known as protein kinase B). It has also been observed that the stimulation of Akt/PKB plays a role in preventing apoptosis [139, 140]. *In vitro* studies have validated that C-peptide stimulates glucose transport in skeletal muscle through a mechanism that does not involve the insulin receptor [141], as well as increasing blood flow to skeletal muscles during physical exercise [142].

#### 3.4.1 Correlation between Sarcopenia and C-peptide

Limited data exist regarding the relationship between fasting plasma C-peptide levels and sarcopenia [26, 95, 98]. In one cross-sectional study, Chen *et al.* [98] indicated that the association between fasting plasma C-peptide levels and the risk of sarcopenia is U-shaped. Specifically, fasting plasma C-peptide was associated with a lower risk of sarcopenia when in the range of 774-939 pmol/L. In another study conducted by Lu *et al.* [95], the levels of C-peptide were found to be lower in participants with sarcopenia and positively associated with body mass index (BMI), Skeletal Muscle Index (SMI), and knee extension strength in the whole sample and those without diabetes. Similarly, other studies by Lin *et al.* [26] and Tanaka *et al.* [143] showed that patients with incident sarcopenia have poor glucose regulation and insufficient insulin secretion and subsequently. Moreover, as C-peptide and insulin decreased in readmitted non-sarcopenia patients, the risk of incident sarcopenia increased. The use of C-peptide has shown a significant ability to prevent muscle weakening in diabetic animals. In an animal model study by Maurotti *et al.* [131], it was demonstrated that the administration of C-peptide in rats has the potential to protect skeletal muscle mass against atrophy caused by type 1 diabetes mellitus. The implications of these findings suggest that the ubiquitin-proteasome system, AMPK, and muscle- specific E3 ubiquitin ligases like Atrogin-1 and Traf6 could be targeted as a viable approach for molecular and clinical intervention in the pathological process of muscle wasting in T1DM.

### 3.5 Adiponectin

Adiponectin, an essential adipokine, is extensively secreted by various cell types, including adipocytes, cardiomyocytes, endothelial cells, and skeletal cells. Its physiological functions are facilitated by receptors that are widely distributed throughout the body, particularly AdipoR1, AdipoR2, and T-cadherin [144, 145]. Several methods have been employed to measure and isolate the biologically active form of the peptide or high-molecular-weight adiponectin, comprised of multiple adiponectin monomers. These techniques include velocity gradient centrifugation, gel filtration chromatography, and polyacrylamide gel electrophoresis. Furthermore, two novel sandwich ELISAs have recently been introduced [146]. Adiponectin's multifaceted biological functions encompass anti-diabetic, anti-atherogenic, anti-inflammatory, and anti-cancer properties [145]. AdipoR1 is most abundantly found in skeletal muscle, whereas AdipoR2 is predominantly present in the liver [147]. Although both AdipoR1 and AdipoR2 are present in skeletal muscles, their relative abundance is influenced by insulin levels, fasting-feeding cycles, and other pathological conditions, potentially mediated through a PI3K/FoxO1 pathway [148]. Overconsumption of nutrients leads to a reduction in adiponectin synthesis and an elevation in the levels of circulating glycosyl phosphatidylinositol-phospholipase D (GPI-PLD). GPI-PLD facilitates the hydrolysis of T-cadherin, resulting in decreased sequestration of adiponectin by responsive tissues, increased adiponectin levels in circulation, and further attenuation of signal transduction mechanisms [149]. The most important known biological role of adiponectin is the regulation of insulin sensitivity in muscle cells, which makes it a central player in T2DM and metabolic syndrome. In humans, adiponectin is secreted by skeletal muscles, where it modulates lipid metabolism through AMPK, p38MAPK, and PPARα pathways [150, 151]. This regulation enhances glucose metabolism *via* the glucose transporter type 4 (GLUT4) receptor and promotes fatty acid oxidation, thereby preserving insulin sensitivity [152, 153]. Recent studies have demonstrated that adiponectin signaling activation may possess a protective role against muscle atrophy by binding to T-cadherin and thereby stimulating muscle regeneration [154, 155].

#### 3.5.1 Correlation between Sarcopenia and Adiponectin

Previous studies have shown inconsistent findings regarding adiponectin levels in individuals with sarcopenia. This could be attributed to different clinical settings, population characteristics, or tools used for defining sarcopenia [156]. Some research suggests that lower levels of adiponectin may contribute to muscle atrophy and decreased muscle volume, indicating an inverse relationship with sarcopenia prevalence [92, 97]. Additionally, adiponectin's impact on skeletal muscle metabolism and its ability to improve insulin sensitivity may offer protection against sarcopenia [157]. In a study using an animal model, Inoue *et al.* demonstrated that the positive effects of physical exercise on muscle metabolism are mediated through adiponectin signaling [158].

However, some studies have shown that sarcopenia is associated with higher adiponectin [159-162]. In a meta-analysis and systematic review conducted by Komici *et al.* [156], seven out of 1,370 initial studies were analyzed. The results revealed a significant increase in adiponectin levels among individuals with sarcopenia [154, 155]. In several studies, the demographics differed in terms of gender. Meta-regression analysis revealed that the difference in the proportion of females between groups with sarcopenia and those without sarcopenia significantly influenced the results. This finding was noteworthy because it had been suggested that women tend to have higher levels of adiponectin compared to men. Additionally, women typically exhibited higher plasma adiponectin levels than men, regardless of their fat mass and BMI [163, 164].

### 3.6 Leptin

The hormone leptin, derived from adipose tissue, is produced in proportion to fat stores. Circulating leptin communicates the body's energy repletion state to the central nervous system (CNS), suppressing food intake and allowing for energy expenditure [165]. When administered externally, leptin has been observed to reduce many physiological changes caused by prolonged fasting. It signals to the brain that energy reserves are replenished, although this signal may be incorrect [166, 167]. Adequate levels of leptin enable energy expenditure for processes like reproduction, tissue remodeling, and growth [168, 169]. Conversely, a lack of leptin signaling, resulting from mutations in leptin or the leptin receptor, leads to increased food intake, reduced energy expenditure, and a phenotype resembling the neuroendocrine starvation response, even in the presence of obesity [170, 171]. Following the identification of leptin, techniques to assess circulating leptin concentrations in humans were promptly developed. Initially, the assays were semi-quantitative, based on the immunoprecipitation/Western blotting technique [172]. The demand for more precise and quantitative procedures was immediately apparent, leading to the development of rapid and precise immunoassays such as RIAs, ELISA, and “in-house” immunoassays [173].

Leptin acts through its receptor (LRb) in the brain to regulate energy balance and neuroendocrine function [169]. LRb signaling, involving STAT3 and other pathways, is necessary for the entirety of leptin's actions [169]. The failure of elevated leptin levels to suppress feeding and induce weight loss in common forms of obesity characterizes a state known as leptin resistance [174]. Various mechanisms, including leptin-stimulated phosphorylation of Tyr985 on LRb and suppressor of cytokine signaling 3, contribute to attenuated leptin signaling and cellular leptin resistance in obesity [175]. Additionally, skeletal muscle may be an important target for leptin as recent studies have shown that leptin is also released from skeletal muscle and its contribution to whole-body leptin production could be significant considering the relatively larger mass of muscle compared to fat [176].

#### 3.6.1 Correlation between Sarcopenia and Leptin

Although the available human data on the correlation between leptin and sarcopenia is limited and inconclusive, findings from studies [177-180] suggest a potential link between lower levels of leptin and an elevated risk of developing sarcopenia. This is attributed to the potential for decreased leptin levels to contribute to muscle atrophy and impaired muscle protein synthesis. Additionally, the complex interaction among leptin, insulin resistance, and inflammation may further exacerbate the progression of sarcopenia (Fig. **3**) [177, 179]. Several animal studies have indicated that leptin plays a crucial role in muscle metabolism in aged mice [181-184]. A recent study has also proposed that higher serum levels of leptin are associated with a reduced risk of sarcopenia [185]. Hamrick *et al.* demonstrate that loss of muscle mass and myofiber size with aging in mice is associated with significant changes in the expression of specific miRNAs and that nutrient-related hormones such as leptin may be able to reverse muscle atrophy and alter the expression of atrophy-related miRNAs in aging skeletal muscle [183]. In another animal model study, Sainz *et al.* [184] investigated changes in muscle gene expression levels related to oxidative stress and inflammatory responses in the presence of leptin deficiency and the effects of administering leptin *in vivo*. The results demonstrated that leptin administration decreased the heightened expression of genes associated with inflammation and muscle loss. In a study by Li *et al.*, elderly individuals with sarcopenia showed significantly higher serum leptin levels and elevated levels of other biomarkers such as serum interleukin (IL)-6, IL-18, and tumor necrosis factor-α (TNF-α), while insulin growth factor-1, insulin, and adiponectin levels were notably lower compared to non-sarcopenic individuals [180]. Yang *et al.* revealed that serum leptin levels were positively correlated with BMI and negatively correlated with SMI (Skeletal Muscle Index), demonstrating a significant association between serum leptin levels and sarcopenic obesity [178]. Furthermore, Kohara *et al.* [186] examined the association between plasma leptin levels and thigh muscle sarcopenia, as well as visceral obesity. Their findings indicated that thigh muscle cross-sectional area (CSA) was negatively correlated with plasma leptin levels, independent of visceral fat area.

### 3.7 Cholesterol and Triglyceride

Cholesterol, a lipid molecule, plays a crucial role in various biological processes, both normal and pathological [187]. Cholesterol serves as a vital structural component in cell membranes, contributing to the biosynthesis, integrity, and functionality of biological membranes, including processes like membrane trafficking and signaling [187, 188]. Additionally, cholesterol is the predominant lipid in lipid rafts, specialized lipid-based structures that regulate the organization and operation of numerous cell signaling pathways [189-191]. Two different classes of lipoproteins perform the function of transporting cholesterol to and from cells. The first class is known as low-density lipoprotein, or LDL, while the second class is referred to as high-density lipoprotein, or HDL [192, 193]. HDL- cholesterol, commonly referred to as “good cholesterol,” is most notably recognized as a crucial player in facilitating the removal of cholesterol from cells and promoting reverse cholesterol transport (RCT), thereby reducing the buildup of foam cells within arterial walls [193]. LDL particles serve as the primary carriers of cholesterol within the circulatory system and their fundamental role lies in transporting cholesterol to the cells [194]. The rigorous regulation of cholesterol homeostasis, encompassing processes such as import, synthesis, export, metabolism, and storage, is crucial given the substantial role of cholesterol in cellular functions. Disruptions in this homeostatic balance have been linked to conditions like atherosclerosis, cardiovascular diseases, and heightened susceptibility to cancer [189].

Moreover, cholesterol is important for insulin-stimulated glucose uptake. The way GLUT4 integrates into cell membranes depends on cholesterol levels, which affect how efficiently glucose is transported [195]. In particular, cholesterol-rich areas in the membrane are essential for glucose uptake, which is vital for cellular metabolism [196]. The significantly elevated cholesterol concentration in the T-tubule membrane of skeletal muscle, surpassing that found in typical cell plasma membranes, underscores its specialized role in promoting glucose uptake and metabolism within muscle tissue [195]. Recent evidence indicates that dysregulated cholesterol levels in the plasma membrane significantly impair GLUT4 trafficking observed in insulin resistance (IR) and T2DM. This dysregulation was demonstrated by the accumulation of cholesterol in skeletal muscle membranes of both animal models and individuals with glucose intolerance, emphasizing the clinical relevance of cholesterol-mediated GLUT4 dysfunction in metabolic diseases [197].

Triglycerides (TGs) are the primary component of animal fat and play a crucial role as carriers of fatty acids [198]. TGs are stored in lipid droplets within skeletal muscle and can be hydrolyzed to generate fatty acids essential for energy production *via* β-oxidation and oxidative phosphorylation (12). The musculoskeletal system retains significant quantities of TG, possessing substantial energetic potential [199]. The noticeable presence of lipid constituents, such as cholesterol and triglycerides, within skeletal muscle tissues suggests their potential involvement in the development and progression of sarcopenia.

#### 3.7.1 Correlation between Sarcopenia and Cholesterol and Triglyceride

Numerous studies have investigated the lipid profile, particularly triglyceride (TG) and cholesterol levels, as potential metabolic indicators distinguishing individuals with sarcopenia from those without [200, 201]. Research using animal models has consistently shown a detrimental relationship between triglyceride content and insulin action [202]. Additionally, the consistent presence of fatty tissue deposition within skeletal muscle, known as myosteatosis, emerged as a hallmark feature of sarcopenia across analyses [203]. In a significant cross-sectional analysis by Chung *et al.*, involving 879 elderly males aged 60 years and older from the 2010-2011 Korean National Health Examination and Nutrition Survey (KHANES), the association between the TG/HDL ratio and sarcopenia was thoroughly examined [203]. The findings revealed a positive relationship between the TG/HDL ratio and the risk of sarcopenia among elderly Korean males. Similarly, another cross-sectional study encompassing 335 participants investigated the interplay among sarcopenia, physical function, arteriosclerosis, and inflammation within a community setting [204]. Utilizing appendicular skeletal muscle mass index (aSMI) measured through bioelectrical impedance analysis for sarcopenia diagnosis, this study unveiled heightened arteriosclerosis risk factors, including elevated blood pressure, cholesterol levels, and high-sensitivity C-reactive protein (hs-CRP), in individuals with sarcopenia compared to their non-sarcopenic counterparts.

Conversely, Wang *et al.* reported contrasting findings in their investigation among community-dwelling Chinese adults [205]. They observed negative associations between sarcopenia occurrence rates and TG levels, while HDL-C levels exhibited a positive correlation. Furthermore, an inverse relationship was noted between sarcopenia occurrence rates and the TG/HDL-C ratio. Similarly, Lin *et al.* documented a negative correlation between the TG/HDL-C ratio and sarcopenia, suggesting a potential marker of improved muscle status [206]. Additionally, in a study involving 72 patients, including 36 with sarcopenia, lower triglyceride levels were observed in sarcopenic individuals [97].

### 3.8 Inflammatory Markers

Inflammation refers to the body's overall defensive response to various harmful stimuli. The diagnosis and monitoring of inflammation involve a thorough assessment that includes clinical observations and laboratory findings. Biochemical markers of inflammation play a crucial role in aiding diagnosis, monitoring progression, and evaluating treatment effectiveness [207]. The evaluation of these markers serves two main purposes: detecting acute inflammation that may indicate specific diseases, and providing insight into treatment response. Additionally, measuring inflammatory markers can serve as a general, though not specific, screening tool for underlying serious diseases [208]. T2DM is closely linked to chronic, low-grade systemic inflammation, which can potentially disrupt glucose and muscle balance [209-211]. Individuals with T2DM often exhibit elevated levels of inflammatory markers like TNF-α, interleukin-6 (IL-6), and CRP [212, 213]. These markers are associated with adiposity and insulin resistance [214] and are elevated in both obese and non-obese individuals with T2DM [213].

TNF-α is a polypeptide hormone with diverse biological functions. Its initial identification as a mediator of tumor necrosis in serum led to further investigations [215], which revealed its ability to induce cytocidal or cytostatic effects on specific tumor cells when cultured in tissue culture settings [216]. Accurately measuring TNF-α is crucial for understanding and investigating related conditions. The typical methods for analyzing TNF-α are based on bioassays or immunoassays. Among the commonly utilized techniques are ELISA, RIA, and bioassays [217]. TNF binds to its receptors TNFR1 and TNFR2, triggering different signaling pathways, including the activation of IKK/NF-kB and MAPK/AP-1 pathways. TNFR1 signaling is robustly activated by both membrane-bound (mTNF-α) and soluble TNF-α (sTNFα), whereas TNFR2 signaling is predominantly stimulated by mTNFα [218]. TNF signaling induces various cellular responses, including apoptosis and necrosis [218, 219]. TNF-α plays a pivotal role in numerous inflammatory conditions such as rheumatoid arthritis [220, 221], inflammatory bowel disease [221], psoriasis [222], ankylosing spondylitis [222], and sarcopenia [223]. Future studies suggest TNF-α may act as a mediator in beneficial processes of host defense, immunity, tissue homeostasis, or in the pathogenesis of infection, tissue injury, and inflammation [224].

IL-6 is a cytokine involved in inflammation, infection responses, and the regulation of metabolic, regenerative, and neural functions [225, 226]. It's synthesized locally in inflammation sites, pivotal in the acute phase response, driving the production of acute phase proteins [227]. The usual methods for studying IL-6 involve immunoassays like ELISA or multiplex immunoassays [228, 229]. IL-6 can bind to trans membrane IL-6R (classic signaling), soluble IL-6R (trans-signaling), or membrane-bound IL-6R (trans-presentation), which leads to the activation of different signaling pathways. These pathways ultimately converge to form a hexameric complex, which includes Glycoprotein (gp) 130 [228, 230]. This IL-6 receptor signaling activates intracellular JAK-MAPK and JAK-STAT3 pathways, which promote IL-6 expression, cell growth, antibody synthesis, and the production of acute phase proteins [228, 230]. Additionally, IL-6 triggers the PI3K/PKB/Akt pathway, which affects the expression of genes essential for cell survival [231]. IL-6 plays a crucial role in inflammation in various diseases, including rheumatoid arthritis [232], cardiovascular disease [233], cancer [234], neurological disorders [235], and muscle wasting associated with sarcopenia [236].

CRP, identified over 70 years ago for its ability to bind to pneumococcal C-polysaccharide, is an acute phase reactant [237]. It is categorized among classical acute-phase proteins because of its biological properties. The liver synthesizes and releases CRP into the bloodstream in response to signals like infection, trauma, or tissue damage triggered by inflammatory cytokines [238]. Structurally, CRP belongs to the pentraxin protein family, which is crucial for pattern recognition within the soluble innate immune system [239]. Standard methods for CRP analysis include the Western blot method [240], biosensors [241], and immunological assays such as ELISA [242-245]. CRP binds to damaged tissue, nuclear antigens, and certain pathogens *via* a calcium-dependent mechanism, bolstering its role within the innate immune system [238]. Similar to immunoglobulin (Ig)G, it activates complement, binds to Fc receptors, and acts as an opsonin for pathogens [244]. Interaction with Fc receptors triggers the production of proinflammatory cytokines, amplifying the inflammatory response [245]. Unlike IgG, which targets specific antigenic epitopes, CRP recognizes altered self and foreign molecules through pattern recognition [244]. As a result, CRP serves as a surveillance molecule for altered self and certain pathogens, initiating early defense mechanisms and activating the adaptive immune system [246]. CRP levels are associated with a spectrum of diseases, including cardiovascular diseases [247], autoimmune disorders [248], infections [246], chronic inflammatory [248, 249] and sarcopenia [250].

#### 3.8.1 Correlation between Sarcopenia and Inflammatory Markers

Several studies have investigated the effects of inflammatory markers on muscle health and strength. Mesinovic *et al.* demonstrated that waist circumference, an indirect measure of visceral adipose tissue (VAT), was negatively associated with muscle strength, quality, and performance in 84 overweight and obese older adults [251]. VAT secretes IL-6 and TNF-α, making it a major contributor to inflammation in Type 2 Diabetes [252]. However, it remains unclear whether these negative associations were driven in part by increased inflammation. Interestingly, IL-6 is also produced by myocytes during exercise, and temporary increases in IL-6 can aid in nutrient mobilization and muscle hypertrophy [253, 254].

On the contrary, IL-6 has been found to trigger muscle atrophy in the tibialis anterior of Sprague-Dawley rats, regardless of systemic inflammation [209]. Park *et al.* discovered that older individuals with Type 2 Diabetes experienced more significant declines in leg muscle mass and strength over three years compared to those without diabetes. Adjusting for cytokine levels, including IL-6 and TNF-α, partially reduced this association, suggesting that inflammation may contribute to diabetes-related muscle decline [255]. Inflammatory markers are not only inversely correlated with muscle mass but also with muscle strength, which is now recognized as the main component of sarcopenia [256].

The secretion of pro-inflammatory cytokines influences anabolic and catabolic pathways in muscle, showing distinct changes in conditions of increased adiposity and age-related muscle wasting [257]. In the contexts of obesity and aging, there is an increase in pro-inflammatory cytokines and CRP in the serum, leading to elevated levels of monocyte chemoattractant protein-1 (MCP-1). MCP-1 binds to the CCLR2 receptor in both muscle and adipose tissues. In adipose tissue, this interaction suppresses the expression of adiponectin and leptin, creating a negative regulatory loop between muscle and adipose tissue that inhibits AMPK, fatty acid oxidation, and glucose uptake. In chronic inflammatory conditions and aging, the heightened serum levels of pro-inflammatory molecules lead to an increase in myostatin. Myostatin, a critical myokine in the muscle-fat interaction [258, 259], acts as a growth factor that inhibits muscle protein synthesis through Smad2/3 signaling and modulates the IGF-1/Akt pathway *via* FOXO transcription factors. It also reduces the activity of GLUT4 and AMPK. This results in decreased glucose uptake and eventual muscle atrophy [260-262] (Fig. **4**). Visser *et al.* reported that among 2,746 generally well-functioning Black and Caucasian men and women aged [27, 65] years in the USA, handgrip strength decreased by 1.1 to 2.4 kg per standard deviation increase in IL-6 concentration [263]. Additionally, TNF-α was negatively associated with grip strength in Black and Caucasian women but not in men [263]. Furthermore, in the English Longitudinal Study of Ageing, CRP showed a negative correlation with handgrip strength in women and chair stand time in both genders [264].

### 3.9 Ketones

Ketone bodies, acetoacetate (AcAc) and beta-hydroxybutyrate (BHB) are metabolic products synthesized in excess in the liver through fatty acid oxidation. These products serve as alternate energy substrates to glucose, particularly during periods of starvation [265]. Upon initiation of ketogenesis, a significant surge in ketone concentration occurs within the millimolar range, predominantly marked by a preferential increase in BHB [266]. Moreover, a fraction of AcAc spontaneously degrades into acetone, constituting the third ketone body, a process associated with the characteristic “fruity” breath odor frequently observed in cases of Diabetic ketoacidosis (DKA) [267]. AcAc and BHB undergo interconversion during the final step of ketogenesis in the liver and the initial step of ketolysis in peripheral tissues [268]. The principal endogenous ketone bodies operate as an alternative energy source in a proportion of approximately 1:10 [269]. In the clinical setting, bedside capillary ketone meters are specifically designed for quantifying BHB levels [270]. While ketone levels can be assessed using both blood and urine samples, the urine ketone stick test provides a cost-effective alternative. Unlike plasma ketone assays, this method does not require specialized equipment or specific training for its implementation [271]. However, it is essential to note that this test cannot detect β-hydroxybutyrate, as it is classified as a hydroxy acid rather than a ketone [272].

A complete absence of insulin characterizes DKA and is primarily observed in individuals diagnosed with type 1 diabetes, although it can also occur in those with type 2 diabetes or gestational diabetes. Enhanced ketogenesis in response to reduced carbohydrate availability or elevated fatty acid levels can lead to ketosis. However, ketoacidosis may develop due to an excessive accumulation of ketone bodies, particularly in cases of unregulated diabetes [273]. Beta-oxidation converts fatty acids into acetyl coenzyme A (CoA), which serves as a substrate in the tricarboxylic acid (TCA) cycle [274]. Under conditions of total insulin deficiency and fatty acid breakdown, the liver produces ketone bodies from excess acetyl CoA due to the limited enzymatic capacity of the TCA cycle [275]. Research indicates that ketone bodies possess appetite-suppressing properties; peripheral injection of 3-hydroxybutyrate (3HB) reduces food intake by enhancing energy metabolism and decreasing the expression of gut cholecystokinin, a peptide associated with appetite suppression [276, 277]. Acetyl CoA serves as the substrate for hepatic ketogenesis, wherein the primary ketone bodies produced are acetoacetate, acetone, and beta-hydroxybutyrate [273]. However, excessive ketone body production may raise concerns about the development of diabetic hyperphagia [278, 279].

Beyond diabetes, ketone bodies play a critical role in conditions such as heart failure [280], Alzheimer's, dementia, and Parkinson's disease. Elevated ketone body levels have been shown to exert beneficial effects on these conditions [281]. One potential area of interest in utilizing ketone bodies is their capacity to mitigate muscle loss in situations of hunger, atrophy, cachexia, or sarcopenia, as well as their potential to enhance muscle protein synthesis and facilitate muscular regeneration following damage [266, 282].

#### 3.9.1 Correlation between Sarcopenia and Ketones

Ketone bodies play a role in metabolic reconfiguration during periods of fasting and diabetes, both of which are associated with muscle loss. This purported “anti-catabolic” effect is based on the ability of ketone bodies to regulate inflammation and cellular biochemistry. This aligns with the proposed evolutionary purpose of ketogenesis, suggesting it protects muscle tissue from degradation during starvation by providing an alternative energy source to gluconeogenesis at a systemic level [266, 283]. Both the ketogenic diet and ketone body infusion have been shown to decrease leucine oxidation, nitrogen excretion, alanine muscle efflux, and indicators of protein catabolism in situations of hunger or acute inflammatory stress [284, 285]. Thomsen *et al.* demonstrated that ketone bodies positively impact muscle protein turnover compared to glucose-induced hyper-insulinemia with lipop- olysaccharide infusion [286]. Thus, substituting insulinogenic substrates with ketones may influence skeletal muscle anabolism in inflammatory conditions. Additionally, exogenous ketones and adherence to a ketogenic diet have proven effective in mitigating muscle wasting in various contexts of cancer cachexia and inflammation-induced atrophy [287, 288].

Evidence also suggests that ketogenic diets or ketone bodies may enhance muscle protein synthesis by activating the mTORC1 pathway. Clinical investigations have shown augmented leucine-induced skeletal muscle protein synthesis with the administration of exogenous ketone bodies [289, 290]. However, the impact of the ketogenic diet or ketone bodies on TOR activity appears to vary significantly depending on the specific context or tissue involved. In a study by Tormita *et al.* in 2020, they explored the effects of SGLT2 inhibition on renal protection in diabetic mice, demonstrating that ketone bodies suppress abnormally increased mTORC1 signaling in the diabetic kidney [291]. The exact processes by which ketone bodies interact with components of the TOR pathway have not been fully understood.

The observed anticatabolic effects are relevant in situations involving dietary restrictions, inflammation, sarcopenia, and cachexia. These findings hold significant implications for the clinical management of age-related diseases, promoting muscle resilience, facilitating rehabilitation, and aiding recovery. The regulation of these activities is believed to involve complex molecular pathways, including NLRP3, HCAR2, NFκB, HDACs, ubiquitination, mTORC1, MEK/ERK, and cellular NADPH pools [292-294].

### 3.10 Renal Function Markers

Numerous markers have been introduced to assess renal function. Serum creatinine (SCr) and blood urea nitrogen (BUN) levels, along with urinalysis, are frequently used indicators for this purpose, although their effectiveness in detecting early stages of renal disorders is limited [295, 296]. Creatinine and urea are byproducts of protein breakdown and are eliminated from the body through various processes. These substances are widely used as diagnostic tools to assess glomerular filtration rate (GFR) [297]. There are several assays available for accurately quantifying creatinine in both urine and serum samples. High-performance liquid chromatography (HPLC) is well-known for its superior sensitivity in measuring creatinine levels. On the other hand, the Jaffe rate reaction, known for its simplicity and cost-effectiveness, is often utilized in clinical contexts [298]. Additionally, both the Enzymatic Method and the Urease-Berthelot Method are commonly recognized as reliable techniques for assessing urea concentrations [299, 300]. It is important to note that variables such as gender, medication usage, dietary habits, and muscle mass may influence the accuracy measurements of these markers [301-304].

Cystatin C is a protein with a molecular weight of 13 kilodaltons. It acts as a cysteine protease inhibitor. The substance is filtered by the glomerulus, then completely reabsorbed and broken down in the proximal tubule without returning to the bloodstream [305, 306]. Cystatin C levels are sensitive to changes in kidney function, especially in mild renal impairment. Furthermore, the half-life of this biomarker is relatively shorter than that of creatinine, making it a more valuable indicator of renal function and the onset of kidney failure [307, 308]. The quantification of cystatin C in serum is commonly done through ELISA, particle-enhanced nephelometric immunoassay (PENIA), and particle-enhanced turbidimetric immunoassay (PETIA) [309]. However, it should be noted that cystatin C levels can be affected by various factors, including thyroid dysfunction, steroid treatment, and inflammation [310, 311].

Fatty acid-binding protein (FABP), discovered in the 1970s, can bind to long-chain fatty acids and certain types of lipids in various parts of the body, such as adipose tissue, intestinal mucosa, muscle, heart, liver, and kidney [312]. There are nine different types of FABPs identified in mammals, including liver-type fatty acid-binding protein (L-FABP) [313]. L-FABP is highly expressed in hepatocytes and the convoluted and straight areas of the proximal tubules in humans. Several clinical studies suggest its potential as a valuable indicator for diabetic kidney disorders [314, 315]. FABPs can be detected using Edman degradation sequencing, matrix-assisted laser desorption, and time-of-flight mass spectrometry [316] and measured using enzyme-linked immunosorbent assay [317]. In addition to renal function, some of these biomarkers are associated with other conditions including tumors, inflammatory diseases, neurological diseases and sarcopenia [318, 319]. A growing body of research indicates a positive association between sarcopenia and kidney disease, particularly diabetic kidney disease [320, 321].

#### 3.10.1 Correlation between Sarcopenia and Renal Function Markers

Serum creatinine serves as a biomarker for assessing muscle protein turnover [36]. However, its concentration is significantly influenced by renal function. To address this limitation, the sarcopenia index (SI), defined as the serum creatinine to serum cystatin C ratio, has been introduced as a tool primarily for evaluating muscle mass [40]. A low SI has been associated with sarcopenia [322] and correlates with atherosclerotic events in sarcopenic patients [323]. The SI was developed to normalize creatinine levels with cystatin C, an additional biomarker reflecting renal function. Nevertheless, the SI's sensitivity and specificity as a biomarker for muscle mass may be inadequate [324]. In a study by Yang *et al.* [325], it was found that the BUN level was significantly higher in sarcopenic patients with T2DM compared to non-sarcopenic patients with T2DM. However, other studies have shown no significant difference in BUN levels between sarcopenic and non-sarcopenic patients with T2DM [94, 97, 82].

In a 2020 study, Tanabe *et al.* demonstrated that the secretion of urinary L-FABP correlates with reductions in muscle strength, muscle weight, and cross-sectional areas of muscle fibers. These findings suggest that urinary L-FABP may serve as a promising biomarker for monitoring the progression of both sarcopenia and diabetic kidney disease in individuals with type 2 diabetes [326]. Additionally, it has been observed that aerobic exercise training can effectively reduce elevated urinary L-FABP levels in healthy middle-aged and older individuals [327]. Studies have also indicated a correlation between decreased physical activity or diminished muscle strength and increased levels of urinary L- FABP [96].

### 3.11 Gamma-Glutamyl Transpeptidase (GGT)

Gamma-Glutamyl Transpeptidase (GGT), also known as E.C.2.3.3.2, is widely distributed across various organisms, including plants and animals. This enzyme is present in both prokaryotic and multicellular eukaryotic species [328]. In humans, GGT can be found in different cell types, particularly in hepatocytes and brain cells [329]. Hepatocytes are the main contributors to to the circulating GGT levels, which can be influenced by genetic and environmental factors [329, 330]. The measurement of GGT levels can be performed using methods approved by the International Federation of Clinical Chemistry (IFCC) and Laboratory Medicine or through medical devices like Siemens ADVIA 2400, Hitachi 7600, Beckman AU5800, and Roche Cobas C501 [331]. Extensive research has revealed GGT's involvement in various diseases, such as Non-Alcoholic Fatty Liver Disease (NAFLD), cardiovascular diseases, and diabetes [332, 333]. Specifically, GGT has been linked to diabetes and insulin resistance [334, 335], and some studies have proposed GGT as a predictive marker for type 2 diabetes [336]. The exact mechanistic link between GGT and diabetes is still unclear, but several hypotheses have been proposed. One hypothesis proposes that the connection between GGT and diabetes could be due to the increased insulin resistance seen in NAFLD, as GGT is an indicator of hepatic cell injury [337, 338]. Furthermore, GGT plays a crucial role in the body's antioxidant mechanisms, and elevated GGT levels indicate higher oxidative stress. It is well-established that oxidative stress can harm pancreatic beta cells, leading to decreased insulin production [339].

#### 3.11.1 Correlation between Sarcopenia and GGT

Previous research has consistently shown a significant positive correlation between elevated levels of GGT and the onset of sarcopenia [340, 341]. GGT has been identified as a key factor that stimulates the production of specific cytokines detrimental to muscle health. Among these cytokines, TNF-α stands out as GGT upregulates its expression. TNF-α is known to inhibit the synthesis of muscle proteins, compromise muscle oxidative function, and impede the process of myogenic differentiation. In addition to its influence on TNF-α, GGT also affects the levels of IL-6, another cytokine associated with muscle degradation. Elevated levels of IL-6, influenced by GGT, promote muscle catabolism and disrupt the normal turnover of muscle proteins [342, 343]. Moreover, GGT plays a pivotal role in glutathione-mediated oxidation pathways [344]. This function is significant as heightened GGT activity exacerbates oxidative damage, leading mitochondrial dysfunction. This impairment subsequently precipitates lipid toxicity, ultimately resulting in muscle degeneration and the progression of sarcopenia [345].

### 3.12 Resistin

Resistin, encoded by the Resistin (RETN) gene [346], is a cysteine-rich polypeptide primarily observed in monocytes/macrophages in humans [347]. This marker has been hypothesized to potentially serve as an inflammatory marker in humans due to its association with immune system cells and its characteristics comparable to cytokines [348]. It plays a pivotal role in the pathogenesis of metabolic disorders and has shown correlations with several health conditions, including obesity, diabetes, insulin resistance, diabetic nephropathy (DN), and NAFLD [349]. In its role as an exclusive secretory factor originating from adipose tissue, Resistin is categorized within the cysteine-rich protein family, displaying prominent expression primarily within white adipocytes and blood cells in murine models [350]. Resistin may play a role in modulating adipocyte differentiation and insulin resistance in the context of T2DM (331). It activates the nuclear factor kappa-B (NF-κB) signaling pathways, leading to the release of TNFα and IL6, which induce insulin resistance. Additionally, resistin's presence in the hypothalamus suggests other functions, including its role in food intake, glucose metabolism, and liver insulin sensitivity [351].

According to a study by Gharibeh *et al.*, serum resistin levels were elevated in patients with T2DM compared to the control group. Moreover, resistin levels were higher in obese individuals with type 2 diabetes than in non-diabetic obese individuals [352]. Another study involving 238 individuals diagnosed with T2DM revealed a significant correlation between serum resistin levels and the progression of DN [353]. The detection of this marker is feasible through human saliva samples and can be quantified utilizing available enzyme immunoassays [354, 355]. Studies have shown that resistin stimulates peripheral and hypothalamic inflammation through the activation of the TLR4 receptor and its downstream signaling. This impairs insulin, adiponectin, and FGF21 signaling in peripheral insulin-sensitive tissues and the hypothalamus [356, 357]. Elevated concentrations of resistin can initiate an inflammatory condition by stimulating the production of proinflammatory cytokines, including IL-1, IL-6, IL-8, and TNF-α, promoting insulin resistance and intensifying oxidative stress [358]. In addition to diabetes mellitus, higher levels of resistin have been associated with other diseases such as inflammatory bowel disease (ulcerative colitis and Crohn’s disease), nonalcoholic fatty liver disease, and sarcopenia [359, 360].

#### 3.12.1 Correlation between Sarcopenia and Resistin

In a study conducted by Ikeda *et al.* (2023), serum resistin levels were evaluated in sarcopenic patients to explore their association with sarcopenic obesity. This cross-sectional investigation included 567 Japanese participants. Multivariate regression analysis demonstrated a positive correlation between elevated serum resistin concentrations and a latent sarcopenic obesity index, as defined by grip strength [361]. Moreover, research has suggested interrelationships among sarcopenic obesity, increased adipose tissue in sarcopenia, elevated resistin levels, and an augmented resistin/IGF-1 ratio [157, 362]. In a separate study from 2022, Gao *et al.* analyzed 235 patients with Chronic Obstructive Pulmonary Disease (COPD) and identified higher serum resistin levels in those with sarcopenia. They proposed that serum resistin levels could be utilized as predictive markers for the onset of sarcopenia [360]. Additionally, aside from facilitating lipid accumulation in muscle cells, resistin has been implicated in promoting cellular metabolism and ATP production. By activating the canonical NF-κB pathway, resistin may also compromise myotube thickness, especially among older individuals [363].

### 3.13 Glucagon-like Peptide-1 (GLP-1)

Glucagon-like peptide-1 (GLP-1) is a peptide hormone consisting of 30-31 amino acids. Intestinal enteroendocrine L-cells produce it in response to nutritional and inflammatory cues, and helps to decrease glucose levels. When GLP-1 binds to the GLP-1 receptor (GLP-1R), it enhances insulin secretion, making GLP-1 agonists useful in diabetes management [364]. Rhere are several methods available for quantifying GLP-1. The Meso-scale discovery method is commonly used for total GLP-1 measurement but has been known to underestimate values. The Bio-Rad kit, specific to human GLP-1, tends to overestimate active GLP-1 levels, leading to significant discrepancies in reported concentrations. Other kits from Alpco, Phoenix, and ELISA kits from USCN LIFE and Gentour are also available [365].

When GLP-1R is activated, adenylate cyclase is activated, leading to cyclic adenosine monophosphate (cAMP) synthesis. This then triggers the PKA-dependent intracellular signaling pathway, ultimately promoting insulin secretion [366]. GLP-1 has been linked to various disorders, such as gallbladder diseases, disrupted insulin secretion, and neurodegenerative conditions [367, 368]. Research on the impact of GLP-1 on human skeletal muscle cells has revealed its role in stimulating myogenesis through a cAMP-dependent network [369]. GLP-1 receptor agonists (GLP-1 RAs) increase GLUT4 expression, glucose uptake, and insulin sensitivity in skeletal muscle [23, 370]. They also enhance microvascular recruitment and promote neuronal recovery after nerve injury, improving muscle health [371-373] (Fig. **2**).

#### 3.13.1 Correlation between Sarcopenia and GLP-1

The influence of GLP-1 on skeletal muscle physiology has been investigated through both *in vitro* and *in vivo* research methodologies. In a controlled study using a mouse model of muscle atrophy induced by dexamethasone, administration of a GLP-1 agonist (exendin-4) showed promising results. This intervention led to significant suppression of genes associated with muscle atrophy, such as myostatin and atrogin-1, ultimately resulting in increased muscle mass and improved functional performance [374]. Moreover, studies have shown that intravenous administration of GLP-1 can improve blood flow by mobilizing microvessels within the skeletal muscle of healthy individuals [375-377]. A retrospective observational study by Osaka *et al.* provided further insights, suggesting potential beneficial effects of GLP-1 agonists in preserving appendicular skeletal muscle mass during hospitalization [378]. Similarly, Xiang *et al.* identified a preventive role of GLP-1 agonists against muscle atrophy in obese mice, mediated through modulation of the SIRT1 pathway [379]. Various other GLP-1 agonists have been investigated in this context. For example, dulaglutide has been found to increase muscle weight and strength by enhancing myofiber size and reducing inflammation through the OPA-1-TLR-9 signaling pathway in aged diabetic mice, thereby counteracting muscle atrophy associated with liver disease [380]. Additionally, a twenty-four-week treatment with liraglutide demonstrated effectiveness in preventing sarcopenia by reducing both fat mass and android fat [381].

## CONCLUSION

The intricate relationship between sarcopenia and diabetes presents a significant challenge in geriatric healthcare. Sarcopenia, characterized by the gradual loss of muscle mass and function, exacerbates issues with glucose metabolism, leading to increased insulin resistance and metabolic complications associated with diabetes. Common underlying mechanisms, including inflammation, oxidative stress, and vascular complications highlight this two-way relationship. Furthermore, circulating biomarkers, which are essential in diabetes management, hold promise for the early detection and monitoring of sarcopenia, offering coast-effective and efficient management strategies. Understanding the connection between sarcopenia, diabetes, and circulating biomarkers has significant implications for enhancing the quality of life and health outcomes for individuals affected by these conditions. The insights gained from this review emphasize the potential value of these biomarkers in paving the way for early detection, intervention, and management strategies that can ultimately lead to improved patient outcomes and quality of life. Tables **1** and **2** further illustrate the relationships and potential biomarkers identified in this review, laying the groundwork for future research and the creation of personalized treatment strategies for sarcopenia and diabetes.

Further investigation is warranted to validate these associations and formulate individualized treatment regimens targeting both sarcopenia and diabetes. Translational medicine is expected to overcome these significant challenges by leveraging recent advancements in the evaluation of circulating biomarkers in diabetes, thereby enabling the development of tailored treatments for the chronic condition of sarcopenia in diabetes.

## AUTHORS’ CONTRIBUTIONS

The authors confirm their contribution to the paper as follows: study conception and design: N.S.; data collection: A.T., V.M. and F.H.; analysis and interpretation of results: A.T. and N.S.; draft manuscript: N.S. All authors reviewed the results and approved the final version of the manuscript.

## Figures and Tables

**Fig. (1):**
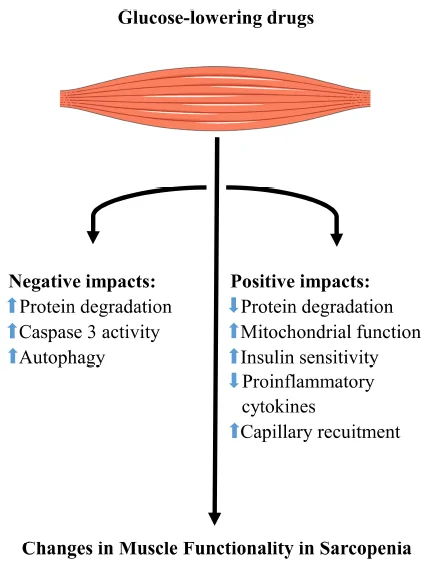
Effect of glucose-lowering medications on sarcopenia. **Note: ** ↑ indicates increase, ↓ denotes decrease [49].

**Fig. (2):**
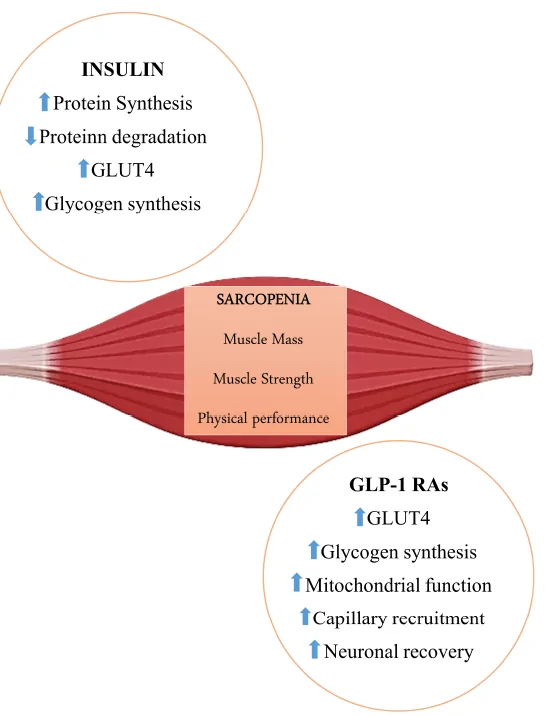
Potential mechanisms of action of two glucose-lowering medications (insulin and GLP-1 RAs) on sarcopenia. GLP-1 RAs refer to glucagon-like peptide-1 receptor agonists. **Note: **↑ denotes an increase, ↓ denotes a decrease. Solid blue, and continue lines represent beneficial effects on sarcopenia [49].

**Fig. (3):**
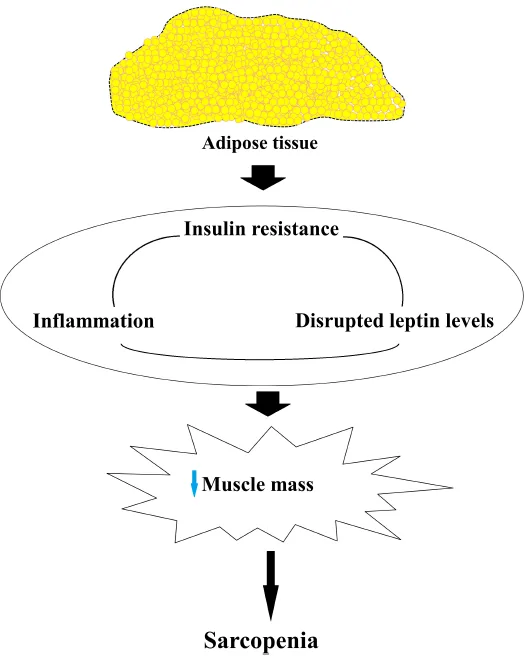
The close interplay between increased adipose tissue, inflammation, insulin sensitivity and development of sarcopenia.

**Fig. (4):**
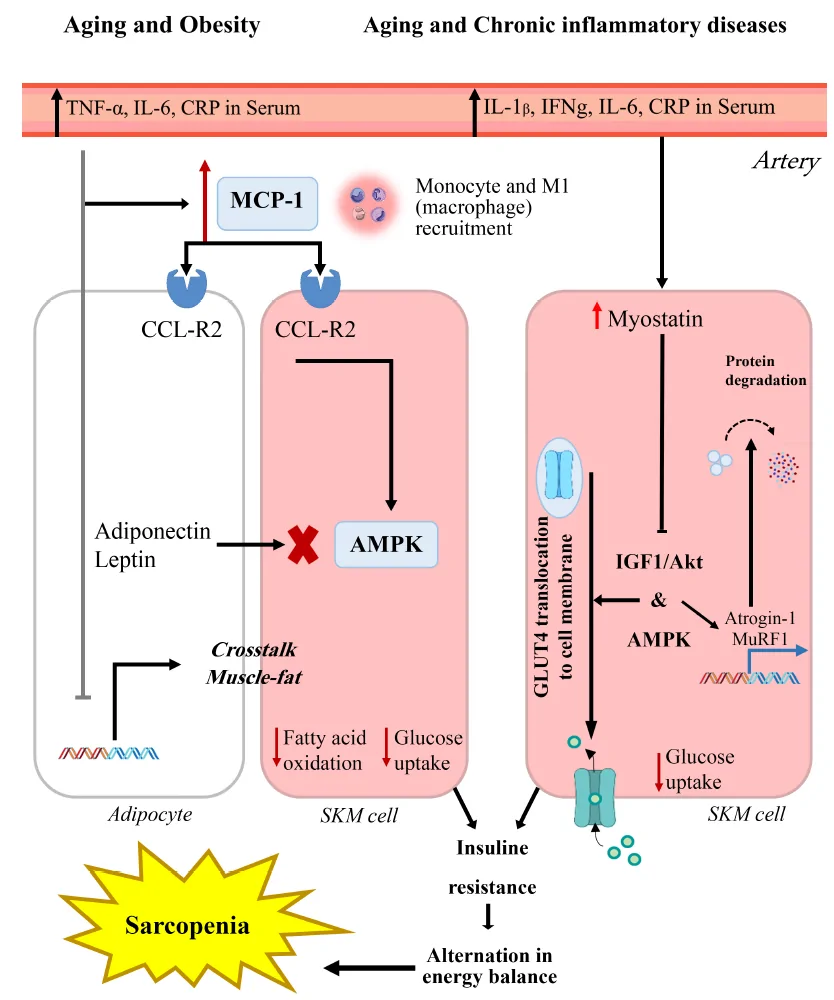
Influence of pro-inflammatory cytokines on muscle metabolism in obesity and aging [262].

**Table 1:** Levels of biomarkers in patients with T2DM and sarcopenia.

**Method**	**Biomarkers Levels**								**References**
	**FBS (mmol/l)**	**HbA1c** (%)	**Cholesterol** (**LDL-HDL**) (mmol/l)	**Triglyceride** (mmol/l)	**Inflammatory Markers**	**Insulin** (pmol/l)	**Creatinine** (µmol/l)	**Others**	
Cross-sectional	7.3 ± 0.5	7.1 ± 0.7	-	-	-	-	-	-	Kaji *et al*. [64]
Cross-sectional	-	6.8 ± 1.3	1.21 ± 0.28 - 2.58 ± 0.9	1.42 ± 0.71	-	-	88.4 ± 70.72	**GGT** (U/l): 34 ± 37	Sung *et al.* [73]
Cross-sectional	-	7.55 ± 1.45	-	-	-		-	-	Ida *et al.* [74]
Cross-sectional	7.7 ± 2.2	7.2 ± 1.0	-	-	-	-	-	-	Hashimoto *et al.* [75]
Cross-sectional	8.6 ± 3.0	7.6 ± 1.2	-	-	-	-	-	**C-peptide** (nmol/l): 0.6 ± 0.5	Okamura *et al.* [76]
Cross-sectional	8.5 ± 0.4	7.7 ± 1.5	-	-	-	-	-	**C-peptide** (nmol/l): 69.9 ± 13.0	Okamura *et al.* [77]
Retrospective (Insulin-treated group)	-	6.9 ± 1.4	2.82 [2.41-3.34] - 1.49 ± 0.42	1.37 [1.02-2.35]	-	-	-	**GGT** (U/l): 35 [22-62]	Bouchi *et al.* [94]
Retrospective (Non-insulin-treated group)	-	7.8 ± 1.4	2.84 [2.22-3.49] - 1.58 ± 0.45	1.15 [0.89-2.02]	-	-	-	**GGT** (U/l): 31 [20-46]	Bouchi *et al.* [65]
Cross-sectional	-	8.1 ± 2.1	2.76 ± 1.05 - 1.2 ± 0.4	1.64 ± 1.16	-	-	-	**-**	Sazlina *et al.* [65]
Cross-sectional	-	7.8 ± 2.8	2.82 ± 0.77 - 1.24 ± 0.28	1.34 [1.02-1.76]	-	-	66.65 [56.25 - 75.70]	**CRP** (mg/dl): 3.02 [3.02-3.13], **BUN** (nmol/l): 6.51 [5.10 - 7.34], Cystatin **c** (mg/dl): 0.93 [0.82 - 1.12]	Cui *et al.* [78]
Cross-sectional	-	7.7 ± 1.3	-	-	-	-	88.4 ± 79.5	**-**	Sugimoto *et al.* [67]
Cross-sectional	-	7.1 ± 0.9	Not measured - 1.6 ± 0.72	1.43 ± 0.52	-	-	-	**-**	Mori *et al.* [27]
Cross-sectional	-	7.3 ± 1.6	-	-	-	-	-	**-**	Ida *et al.* [79]
Cross-sectional	-	9.5 ± 2.1	2.75 ± 0.95 - 1.19 ± 0.43	1.66 [1.1 -2.44]	-	-	-	**C-peptide** (nmol/l): 0.6 ± 0.4, **Adiponectin** (µg/ml): 7.3 [4.6 - 11.3]	Murai *et al.* [80]
Cross-sectional	-	7.1 ± 1.0		-	-	-	70 ± 26	**BUN** (nmol/l): 5.78 ± 1.96, **Cystatin c** (mg/dl): 0.9 ± 0.3	Osaka *et al.* [81]
Retrospective	-	8.1 ± 2.1	2.74 ± 0.78 - 1.59 ± 0.53	1.12 [0.82-1.76]	-	-	-	**GGT** (U/l): 30 [20-48]	Fukoda *et al.* [82]
Cross-sectional	-	7.9 ± 1.9	Not measured -1.33 ± 0.46	-	-	-	79.56 ± 35.36	**CRP** (mg/dl): 4.85 [0 - 9.7]	de Freitas *et al.* [83]
Cross-sectional	-	7.4 ± 1.0	-	-	-	-	-	**-**	Ida *et al.* [84]
Cross-sectional	-	7.02 ± 1.00	2.46 ± 0.71 -1.46 ± 0.4	1.22 ± 0.66	-	-	-	**BUN** (nmol/l): 6.6 ± 2.8, **GGT** (U/l): 29.2 ± 27.1	Fukuoka *et al.* [93]
Cross-sectional	-	8.1 ± 2.0	-	-	-	-	-	**-**	Liccini *et al.* [85]
Cross-sectional	-	7.44 ± 0.96	2.55 ± 0.79 -3.0 ± 2.08	-	-	-	-	**-**	Murata *et al.* [86]
Randomized controlled trial	-	8.1 ± 1.1	1.91 ± 0.97 -1.31 ± 0.41	1.19 ± 0.48	-	-	-	**-**	Chien *et al.* [87]
Cross-sectional	-	-	-	1.24 ± 0.44	-	-	-	**Adiponectin** (µg/ml): 6.0 ± 10.5, **CRP** (mg/dl): 1.01 ± 4.99	Can *et al.* [97]
Prospective	-	-	-	-	**TNF-α** (pg/ml): 93.61 ± 83, **IL-6** (pg/ml): 1.28 ± 0.68	22.15 ± 17.5	-	**Adiponectin** (µg/ml): 3.53 [1.63-7.39], **Leptin** (ng/ml): 11.62 [6.23-25.49]	Li *et al.* [92]
Cross-sectional	-	8.2 ± 1.8	2.19 ± 0.87 -1.12 ± 0.3	1.66 ± 0.61	-	-	-	**-**	Alfaro-Alvarado *et al.* [88]
Cross-sectional	-	7.9 ± 1.5	-	-	-	-	-	**-**	Pechmann *et al.* [89]
Cross-sectional	-	9.14 ± 2.54	-	-	-	-	-	**C-peptide** (nmol/l): 0.71 ± 0.43	Chen *et al.* [98]
Randomized controlled trial (Exploratory study)	5.35 ± 1.37	-	-	-	-	26.04 ± 16.20	-	**C-peptide** (nmol/l): 0.44 ± 0.2, **Leptin** (ng/ml): 8.92 ± 9.49	Lu *et al.* [94]
Randomized controlled trial (Exploratory study)	6.23 ± 1.70	-	-	-	-	16.16 ± 14.36	--	**C-peptide** (nmol/l): 0.3 ± 0.12, **Leptin** (ng/ml): 10.96 ± 9.27	Lu *et al.* [94]
Longitudinal cohort study	-	-	1.50 ± 1.02	-	-	-	66.46 ± 27.66	**CRP** (mg/dl): 7.89[3.77 -18.34]	Lin *et al.* [26]
Cross-sectional	-	8.88 ± 2.41	-	-	**TNF-α** (pg/ml): 1.43 ± 0.55, **IL-6** (pg/ml): 1.20 ± 0.27		-	**-**	Hou *et al.* [91]
Cross-sectional	5.1 [4.7-5.4]	-	3.4 ± 0.8 - 1.6 ± 0.36	0.97 [0.68- 1.33]	-	-	61 ± 13.2	**BUN** (nmol/l): 5.39 ± 1.28, **Urinary L-FABP** (μg/g creatinine): 1.67 [1.08-2.72], **Cystatin c** (mg/dl): 0.75 [0.67-0.81]	Kosaki *et al.* [96]
Cross-sectional	6.75 [5.86 - 10.34]	7.85 [6.75 -10.03]	2.47 [1.61 - 3.10] - 1.25 [0.93 - 1.38]	1.43 [1.58- 2.01]	-	-	-	**-**	Ma *et al.* [90]

**Note: **Normally distributed data are expressed as mean ± standard deviation, while non-normally distributed data are represented as median and interquartile range.

**Table 2:** Reference ranges for biomarkers in healthy adults and adolescents.

**S.No.**	**Biomarker**	**Normal Limits [Unit]**	**References**
1	FBS	70-100 [mg/dL] or 3.9 - 5.6 [mmol/l]	[99]
2	HbA1c	Less than 5.6 [%] considered as normal and higher levels considered as prediabetic and diabetic	
3	LDL	less than or equal to 100 [mg/dL] or 2.58 [mmol/L] considered as optimal	
4	HDL	greater than or equal to 40 [mg/dL] or 1.04 [mmol/L] considered as optimal	
5	Triglyceride	less than 150 [mg/dL] or 1.69 [mmol/L] considered as normal	
6	Insulin	6-27 [µIU/ml]	
7	Creatinine	Female: 0.5-1.2 [mg/dL] or 44.2-106.08 [µmol/L] Male: 0.6-1.3 [mg/dl] or 53.04-114.92 [µmol/L]	
8	GGT	7-50 [IU/L]	
9	C-peptide	0.9-7.1 [ng/dl] or 0.3 to 2.3 [nmol/L]	
10	CRP	0.0-0.3 [mg/dL] or 0.0-3.0 [mg/L]	[99]
11	BUN	7-23 [mg/dL] or 2.45-8.05 [mmol/L]	
12	Cystatin C	0.5-1.0 [mg/L]	
13	Adiponectin	People with a BMI less than 25: Female: 5-37 [µg/ml] Male: 4-26 [µg/ml] People with a BMI of 25 to 30: Female: 5-28 [µg/ml] Male: 4-20 [µg/ml] People with a BMI over 30: Female: 4 to 22 [µg/ml] Male: 2-20 [µg/ml]	
14	Leptin	Male: 0.5-12.7 [ng/ml] Female: 3.9-30.0 [ng/ml]	
15	Urinary L-FABP	Less than or equal to 8.4 [μg/gCr]	[100]
16	TNFα	60-90 [pg/dl]	[101]
17	IL-6	Less than 10 [pg/dl]	[102]

## LIST OF ABBREVIATIONS

DM: Diabetes Mellitus
TG: Triglyceride
T2DM: Type 2 Diabetes Mellitus
AGE: Advanced Glycation end Products
IGF-1: Insulin-like Growth Factor 1
GLP-1: Glucagon-like Peptide-1
FPI: Fasting Plasma Insulin
IR: Insulin Resistance
DN: Diabetic Nephropathy
NAFLD: Non-alcoholic Fatty Liver Disease
GGT: Gamma-glutamyl Transpeptidase
L-FABP: Liver-type Fatty Acid-binding Protein
ELISA: Enzyme-linked Immunosorbent Assay
PETIA: Particle-enhanced Turbidimetric Immunoassay
AcAc: Acetoacetate
Ig: Immunoglobulin
IL-6: Interleukin-6
LRb: Leptin Acts Through its Receptor
BMI: Body Mass Index
GPI-PLD: Glycosyl Phosphatidylinositol-phospholipase D
AMPK: AMP-activated Protein Kinase
RIA: Radioimmunoassay
CLIA: Chemiluminescence Immunoassay
TBC1D1: Domain Family Member 1
HbA1c: Hemoglobin A1c
IPGTT: Intraperitoneal Glucose Tolerance Test
OGTT: Oral Glucose Tolerance Test
FOXO: Fork Head Box O
BUN: Blood Urea Nitrogen
hs-CRP: High-sensitivity C-reactive Protein
FPG: Fasting Plasma Glucose
HOMA: Homeostatic Model Assessment
MTT: Meal Tolerance Test
OGTT: Oral Glucose Tolerance Test
IPIST: Intraperitoneal Insulin Sensitivity Test
GLP-1R: GLP-1 Receptor
IPGTT: Intraperitoneal Glucose Tolerance Test
cAMP: Cyclic Adenosine Monophosphate
COPD: Chronic Obstructive Pulmonary Disease
RETN: Resistin
IFCC: International Federation of Clinical Chemistry
SI: Sarcopenia Index
PENIA: Particle-enhanced Nephelometric Immunoassay
HPLC: High-performance Liquid Chromatography
BHB: Beta-hydroxybutyrate
Gp: Glycoprotein
KHANES: National Health Examination and Nutrition Survey
CNS: Central Nervous System
GLUT4: Glucose Transporter Type 4
ERK1/2: Extracellular Signal-regulated Kinase 1/2
T1DM: Type 1 Diabetes Mellitus
ELISA: Enzyme-linked Immunosorbent Assay
TBC1D4: TBC1 Domain Family Member 4
LC-MS/MS: Liquid Chromatography with Tandem Mass Spectrometry
TIR: Time in Range
IPIST: Intraperitoneal Insulin Sensitivity Test
MTT: Meal Tolerance Test
MCP-1: Monocyte Chemoattractant Protein-1
SCr: Serum Creatinine
TNF-α: Tumor Necrosis Factor-α
